# Internal fossil constraints have more effect on the age estimates of crown Palaeognathae than different phylogenomic data type

**DOI:** 10.3389/fbinf.2025.1563786

**Published:** 2025-08-07

**Authors:** Alexandre Pedro Selvatti, Naoko Takezaki

**Affiliations:** ^1^ Department of Zoology, Biology Institute, Rio de Janeiro State University - UERJ, Rio de Janeiro, Brazil; ^2^ Laboratory of Life Sciences, Faculty of Medicine, Kagawa University, Kagawa, Japan

**Keywords:** aves, time tree, fossil priors, internal constraints, relaxed molecular clock

## Abstract

Palaeognathae is an ancient bird lineage that includes the volant tinamous and six flightless lineages: ostrich, rhea, cassowary, emu, kiwi (extant) and moa, elephant bird (extinct). Over the past decade, a consensus has emerged on the relationships within the group. In this consensus, the ostrich branch splits first, followed by rheas, a clade containing tinamou and moa and a clade with the emu and cassowary sister to the kiwi and elephant bird. However, the timing of the origin of these major clades remains uncertain. In phylogenomic studies, the origin of the crown Palaeognathae is typically dated to the K–Pg boundary (∼66 Ma), though one study suggested a younger Early Eocene age (∼51 Ma). This discrepancy might result from the number and position of fossil priors (calibration strategies) or by differences in genomic regions sampled (data types). We investigated the impact of calibration strategies and data types on the timing of the Palaeognathae root using genomic sequences from nuclear (noncoding [CNEE and UCE] and coding [first and second codon positions]) and mitogenomic datasets. The nuclear dataset included 14 Palaeognathae species (13 extant and the extinct moa), while the mitogenomic included 31 species, covering all extant and extinct lineages. The datasets were analyzed with and without internal calibrations. The age estimates were more influenced by calibration strategy than data type, although some nuclear data (CNEE) produced substantially younger ages except for the Casuariiformes node, whilst another dataset (PRM) from a previous study estimated younger ages for Casuariiformes compared to the other datasets. Nevertheless, our results consistently placed the origin of crown Palaeognathae around the K–Pg boundary (62–68 Ma), even when using the original dataset that produced the Eocene age. These findings demonstrate that multiple internal calibrations yield consistent results across different sequence types and taxon schemes, providing robust estimates of the crown Palaeognathae age. This improved timing enhances our understanding of the early evolutionary history of this clade, particularly regarding the placement of enigmatic Paleocene fossils, such as Lithornithidae and *Diogenornis*, which in this timeframe can be assigned to internal branches within the crown Palaeognathae.

## 1 Introduction

Geological time forms the backdrop of biological diversification in Evolutionary Theory. Until 60 years ago, the temporal study of biodiversity was limited to fossils, but with the introduction of the molecular clock hypothesis, the concept of an evolutionary timescale was expanded to encompass both hereditary material and the peptide products it encodes (Kumar, 2005). This enabled the reconstruction of the tree of life with unprecedented detail, as living branches became sources of chronological data. As one of the most studied animal groups, birds significantly contribute to our understanding of biodiversity, including genomics, ecology, and biogeography (Jarvis et al., 2014; Zhang et al., 2014b; 2014a; Claramunt and Cracraft, 2015; Stiller et al., 2024). With over ten thousand species, living birds (Neornithes) combine a rich genetic diversity with a significant fossil record that extends to the Late Cretaceous (Mayr, 2016a), making them a celebrated branch in the efforts to assemble the time tree of life.

**FIGURE 1:**
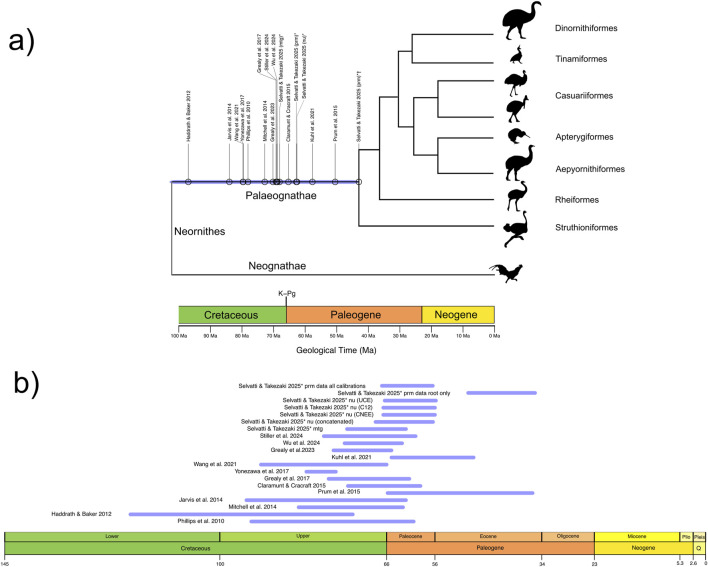
Divergence times estimated for the crown Palaeognathae root using phylogenomic data. **(a)** Blue line indicates the mean ages distributed along a unified confidence interval for the Palaeognathae root (see also Table 1). The gray color on the vertical line at the Neornithine root highlights the age uncertainty for that node. Branch lengths within Palaeognathae display relationships among the major clades and do not reflect divergence times. Abbreviations indicate data type: mtg, mitogenomic; nu, our main nuclear dataset; prm, nuclear dataset that estimated the Eocene age for crown Palaeognaths. Symbols are as follows: *, this study; †, PRM dataset without ingroup Palaeognathae fossil calibrations. Geologic Time units in Million years ago (Ma). **(b)** Confidence intervals of each individual study for the estimated age of the crown Palaeognathae root for details, see Table 1.

Morphological and genomic data split the Neornithes between the Neognathae and the Palaeognathae clades (Livezey and Zusi, 2007; Prum et al., 2015). Although palaeognaths comprise less than 1% of neornithine species, they include the largest and heaviest birds to ever live (Crouch and Clarke, 2019). Featuring gigantism, palaeognaths encompass several flightless, continentally endemic lineages from the Southern Hemisphere. The unique phylogenetic, morphological, and biogeographic traits make palaeognaths a key group for understanding early neornithine evolution in space, form, and time (Harshman et al., 2008; Widrig and Field, 2022). Most nuclear and mitogenomic data support an early divergence of ostriches (Struthioniformes) followed by rheas (Rheiformes). The tinamous (Tinamiformes) plus the recently extinct moas (Dinornithiformes) split next and the remaining palaeognaths form a clade comprising emus and cassowaries (Casuariiformes) sister to kiwis (Apterygiformes) and the recently extinct elephant birds (Aepyornithiformes) (Figure 1a; Mitchell et al., 2014; Yonezawa et al., 2017; Takezaki, 2023).

Yet, a clear-cut picture on palaeognaths early evolution remains tentative as phylogenomic studies disagree on the age of the most recent common ancestor of extant (crown group) Palaeognathae. The most common method to estimate the divergence times between lineages using molecular data is the Bayesian relaxed clock (dos Reis et al., 2015). This method adequately accommodates the uncertainty in both evolutionary rates and fossil dates, which are incorporated as prior probability distributions [calibration priors; see Glossary]. The information in the observed data and the priors are integrated to convert the sequence differences into absolute geological times (Yang, 2006; Ho and Phillips, 2009). Hence, the selection of fossils as calibration priors must follow rigorous criteria (Parham et al., 2012) so the resulting time tree is robust and consistent across datasets. Virtually all phylogenomic studies with different gene and taxon coverage agree that the crown Palaeognathae split occurred between the Late Cretaceous and the Earliest Paleogene (K–Pg age) (Figure 1b; Jarvis et al., 2014; Mitchell et al., 2014; Claramunt and Cracraft, 2015; Grealy et al., 2017; Yonezawa et al., 2017; Stiller et al., 2024; Wu et al., 2024).

However, one study (Prum et al., 2015) deviates markedly by suggesting an Early Eocene age for crown palaeognaths around 51 Ma (Figure 1). Although that study followed rigorous criteria for fossil prior selection, a striking pattern is observed in the number and position of fossil priors [calibration strategies] (Table 1). In the calibration strategy that resulted in the Eocene age, all fossil-based priors were restricted to the Neognathae clade (Prum et al., 2015). In contrast, the K–Pg age is consistently recovered in studies that included at least a fossil-based calibration at the neornithine root, and most studies included at least one calibration within Palaeognathae, whereas none was used to generate the Eocene age (Table 1). The impact of calibration strategies in Bayesian molecular dating has been extensively evaluated. Simulated and empirical data show that calibration priors at or near the root produce low error and high precision (Duchêne et al., 2014; Mello and Schrago, 2014; Carruthers et al., 2020). Those studies also demonstrated that the lack of fossil priors on deep nodes produces undesirable effects such as inconsistent and unrealistic age estimates. The Eocene age was obtained with no fossil priors to any of the three deepest avian nodes, namely, Neornithes, Galloanseres and Neoaves. Thus, as the common ancestor of Palaeognathae represents a deep node, the exclusion of time priors for this clade and for the neornithine root may have biased divergence time estimates, potentially leading to an underestimation of its divergence time. Alternatively, as taxonomic and marker sampling varied extensively across previous studies (Table 1), the resulting discrepancies in divergence time estimates may reflect differences in evolutionary rates across lineages, genes, or genomic regions.

**TABLE 1:** Phylogenomic studies that estimated the divergence times of Palaognathae lineages. Number of loci are given for mitochondrial individual genes (mt), complete or nearly complete mitogenomes (mtg) and nuclear (nu).

Study	Number of species ingroup	Number of loci	Matrix length (bp)	Calibration for neornithes root	Number of calibrations ingroup	Ingroup calibration specimen(s)	Mean age (Ma)	95% confidence interval (Ma)
Phillips et al. (2010)	14	mtg	14,190	Yes	2	*Emuarius*; *Diogenornis fragilis*	78.1	60.4–94
Haddrath and Baker (2012)	16	10 mt; 27 nu	9,909; 31,685	Yes	1	*Emuarius*	97	73–119
Mitchell et al. (2014)	10	mtg	14,988	Yes	1	*Emuarius*	72.8	62.6–84.2
Jarvis et al. (2014)	2	14,536 nu	41,800,000	Yes	1	MACN-SC-1399	84	62–95
Prum et al. (2015)	9	259 nu	394,684	No	0	-	50.5	35.8–65.8
Claramunt and Cracraft (2015)	7	2 nu	4,092	Yes	2	*Diogenornis fragilis*; *Emuarius*	65.3	59–74
Grealy et al. (2017)	16	mtg; 154 nu	27,116	Yes	2	*Diogenornis fragilis*; *Emuarius*	69.2	61.3–78
Yonezawa et al. (2017)	22	mtg; 663 nu	871,449	Yes	1	*Emuarius*	79.6	76.5–82.6
Wang et al. (2022)	15	mtg; 4,387 nu	393,181,149	Yes	0	-	79.7	66–92
Kuhl et al. (2020)	11	nu	2,584,785	Yes	1	MACN-SC-1399	57.7	48–65
Grealy et al., 2023	23	mtg; 154 nu	27,699	Yes	4	*Diogenornis fragilis*; *Emuarius*, MACN-SC-1399; *Proapteryx*	70.2	65–77
Wu et al. (2024)	6	25,460 nu	20,652,290	Yes	0	-	68.8	62.8–74.7
Stiller et al. (2024)	19	63,430 nu	63,430,000	Yes	4	*Emuarius*; *Opisthodactylus horacioperezi*; MLP 87-XI-20–3; AMNH FAM 9151	69	60–79
Selvatti and Takezaki 2025^a^	31	mtg	14,307	Yes	4	*Palaeophasianus meleagroides*; *Opisthodactylus horacioperezi*; MACN-SC-3610; *Emuarius*	68.1	62–74.2
Selvatti and Takezaki 2025^a^	14	19,298 nu (concatenated)	11,187,881	Yes	4		62.4	56.4–68.3
Selvatti and Takezaki 2025^a^	14	12,561 nu (CNEE)	4,504,498	Yes	4		61.2	56–66.7
Selvatti and Takezaki 2025^a^	13	5,374 nu (C12)	4,797,876	Yes	4		61.1	56–66.7
Selvatti and Takezaki 2025^a^	14	1,363 nu (UCE)	1,885,507	Yes	4		60.9	55.8–66.4
Selvatti and Takezaki 2025^a^ prm data^b^	9	259 nu	394,684	Yes	0		42.4	35.6–49.4
Selvatti and Takezaki 2025^a^ prm data^c^	9	259 nu	394,684	Yes	3	*Palaeophasianus meleagroides*, MACN-SC-3610, Emuarius	62.4	56.7–67.2

Mtg data is around 15,000 bp. Time units are million years ago (Ma) and rounded up using two decimal places as reference. Confidence Interval is measured as 95% Highest Posterior Distribution (HPD). Superscript symbols as follows.

^a^ This study.

^b^ Prum et al., 2015 data without internal calibrations.

^c^ Prum et al., 2015 data with internal calibrations.

We investigated if the conflict between the K–Pg and the Eocene age of crown Palaeognathae was caused by differences in fossil calibration strategies or phylogenomic data type. We used complete mitogenomes that included living and extinct species, resulting in the most comprehensive mitogenomic time tree estimated for Palaeognaths to date (Figure 1; Table 1). The nuclear dataset comprises over 10 million base pairs (bp) of non-recombinant loci from various genomic regions across all extant lineages and the extinct moa (Takezaki, 2023), and is used here for the first time to estimate divergence times. We tested whether a dataset tailored to resolve palaeognath relationships could also produce robust divergence time estimates under varying loci and fossil calibration strategies. We also reanalyzed the dataset of Prum et al. (2015) using different fossil calibration strategies and discussed how these affect our main results.

## 2 Methodological details

### 2.1 Molecular data assembly

We assembled a wide diversity of molecular markers from previously published studies. All species names from each study were updated according to the International Ornithological Committee v. 14.2 (Gill et al., 2024). The markers represent four distinct classes of genomic markers, namely, conserved non-exonic elements, coding sequences, ultraconserved sequences, and mitochondrial genomes. Each dataset assembly is detailed next and summarized in Table 1.

First, we created a mitogenomic dataset [MTG] that consisted of complete or nearly complete mitochondrial genomes available at NCBI (https://www.ncbi.nlm.nih.gov/genbank/). Priority was given for RefSeq sequences and the longest sequence with the least number of undetermined bases. We extracted and concatenated all 13 protein coding genes and the ribosomal 12S and 16S genes, resulting in 31 Palaeognathae species and 14,307 bp. The species and accession numbers of all mitogenomes used in this study were *Aepyornis hildebrandti* [KJ749824], *Aepyornis maximus* [OP413809], *Anomalopteryx didiformis* [MK778441], *Apteryx australis* [MN356385], *Apteryx haastii* [NC_002782], *Apteryx mantelli* [AY016010], *Apteryx owenii* [GU071052], *Apteryx rowi* [MN998652], *Casuarius bennetti* [AY016011], *Casuarius casuarius* [MN356153], *Crypturellus cinnamomeus* [NC_052825], *Crypturellus soui* [MN356154], *Crypturellus tataupa* [AY016012], *Crypturellus undulatus* [NC_052774], *Dinornis giganteus* [AY016013], *Dromaius baudinianus* [NC_045365], *Dromaius novaehollandiae* [MN356172], *Emeus crassus* [AY016015], *Eudromia elegans* [AF338710], *Mullerornis agilis* [KJ749825], *Mullerornis modestus* [OP413795], *Nothocercus julius* [MN356379], *Nothocercus nigrocapillus* [MN356380], *Nothoprocta ornata* [MN356381], *Nothoprocta pentlandii* [MN356382], *Nothoprocta perdicaria* [MN356428], *Rhea americana* [NC_000846], *Rhea pennata* [NC_002783], *Struthio camelus* [AF338715], *Tinamus guttatus* [MN356150], *Tinamus major* [NC_002781] (Härlid et al., 1998; Cooper et al., 2001; Haddrath and Baker, 2001; Phillips et al., 2010; Mitchell et al., 2014; Cibois et al., 2020; Feng et al., 2020; Lan and Xu, 2020; Grealy et al., 2023; Edwards et al., 2024). The RefSeq mitogenome of the chicken *Gallus gallus* [NC_040902] (Miao et al., 2013) was used as outgroup.

We used a nuclear dataset from Takezaki (2023) [TKZ]. This dataset includes all extant (ostrich, rhea, cassowary, emu, kiwi and tinamou) and one extinct (moa) lineages and comprises the noncoding data originally from Cloutier et al. (2019) and the coding data from Sackton et al. (2019). However, extensive gene tree heterogeneity was observed in the original datasets (Cloutier et al., 2019; Sackton et al., 2019), suggesting potential bias from estimation errors or other misrepresentative evolutionary signals. To mitigate this, loci likely to introduce such biases were identified and filtered following the criteria outlined below (Takezaki, 2023). First, loci were excluded if the branch lengths within palaeognaths in their gene trees were more than five times longer than those in the concatenated sequence tree. Loci showing significant evidence of positive selection or recombination between palaeognath lineages and *Gallus* were also removed. Importantly, during the filtering process, no species were excluded, and no tip pruning was applied, ensuring all species were retained as in the original datasets. By retaining all species and focusing on molecular data types, we ensured that the filtered loci had more homogeneous composition and substitution rate variation, thus low probability of saturation, while preserving all palaeognath nodes necessary for reliable divergence time estimation. The filtered datasets yielded a robust topology with significantly reduced sequence heterogeneity and were used as the primary nuclear genomic source for estimating divergence times in palaeognaths. Noncoding data (conserved non-exonic elements: CNEEs, 12,561 loci and ultraconserved elements: UCEs 1,363 loci) contained 14 Palaeognathae species and coding data (the first and second codon positions: C12, 5,374 loci) for 13 Palaeognathae species were used. The third codon positions and intron data were excluded from the analyses as they have the highest GC content and long branch lengths compared to other data (Takezaki, 2023). All 19,298 loci were concatenated into a supermatrix of 15,610,067 bp and cleaned of poorly aligned sites and highly divergent regions using Gblocks (Castresana, 2000), resulting in our final TKZ matrix with 11,187,881 bp. Finally, we reanalyzed the original data from Prum et al. (2015) [PRM], which consisted of nine Palaeognathae species and 259 coding and non-coding regions (around 400 Kbp) obtained from anchored hybrid enrichment (Lemmon et al., 2012). The alignments generated in this study (MTG and TKZ) including all species sampled for each dataset are available as Supplementary Material S1.

### 2.2 Fossil specimens

We selected five fossil specimens that rigorously fit the criteria to be used as calibration priors. We opted for the vouchered oldest specimens with unambiguous locality and stratigraphy, and an apomorphy-based diagnosis that is consistent between morphological and molecular datasets in phylogenetic context (Parham et al., 2012). Data for all five fossil specimens, including stratigraphic age, phylogenetic placement justification, and references, are detailed in Table 2 and summarized below.

**TABLE 2:** Fossil specimens with phylogenetic justifications used for estimation of divergence times. Age and age uncertainty probabilities (minimum and maximum soft bounds) and prior configuration as used in MCMCTree.

Specimen	Stratigraphy	References for description, stratigraphic dating and phylogenetic placement	Calibrated node	Phylogenetic placement justification and key apomorphic diagnosis	Hard minimum age (Ma)	Soft maximum age (Ma)	Calibration in MCMCTree (function, location, scale, alpha)
*Asteriornis maastrichtensis*	CBR-Romontbos Quarry, Valkenburg Member Formation (Late Maastrichtian), Eben-Emael, Liège, Belgium	Field et al., 2020; Mayr 2022; Crane et al., 2024	Neornithes	Stem-galloanserine with key mandiblar apomorphies of Galloanseres, and derived characters in the femur are shared exclusively with Neognathae. *Asteriornis*indicates that the Neornithes ancestor had already split between Neognathae and Palaeognathae by the Latest Cretaceous	66	86.5	SN (66.5, 7, 10)
*Palaeophasianus meleagroides*	Bighorn Basin, Willwood Formation (Early Eocene), Wyoming, United States	Wetmore 1933; Mayr 2016a; Mayr 2019	Crown Palaeognathae	Hindlimb bones share derived morphology with other stem Struthioniformes such as Palaeotididae, such as dorsal surface of tarsometatarsus strongly marked by sulcus extensorius, hypotarsus proximodistally elongated with a long medial crest, elongated tarsometatarsus and distinct notch in distal rim of condylus medialis of the tibiotarsus	56	66	SN(56, 4, 10)
*Opisthodactylus horacioperezi*	Colhuehuapian SALMA [South American Land Mammal Age] Chichínales Formation (Early Eocene), Rio Negro Province, Patagonia, Argentina	Noriega et al., 2017; Agnolin and Chafrat 2015; Picasso et al., 2022	Crown Rheidae	Derived hindlimb morphology shared with the clade in Rheidae that includes other species of *Opisthodactylus*(extinct) and the extant *Pterocnemia*, such as tarsometatarsus with dorsoplantarly flattened shaft and divergent distal trochleae; and tibiotarsus with medial edge of crista cnemialis medialis forming a short ridge extending distally on shaft and prominence for attachment of internal ligament continuous with attachment of transverse ligament	23	56	SN (23, 11, 10)
MACN-SC-3610	Santa Cruz Formation, Monte Observatión, Patagonia, Argentina	Bertelli and Chiappe 2005; Bertelli et al., 2014; Bertelli 2017	*Tinamus + Crypturellus*	Derived features of proximal coracoid morphology such as absence of a cranially projected processus acrocoracoideus and presence of a foramen on the proximal margin of cotyla scapularis supports a well resolved position as sister to the *Crypturellus*genus	20.4	56	SN (20.4, 15, 10)
*Emuarius gidju*	Etadunna Formations, Leaf Locality (UCMP V-6213), WipaJiri Formation, Lake Ngapakaldi, Etadunna Station, Australia	Boles 1992; Worthy et al., 2014	Crown Casuariiformes	Phylogenetic position of *E*. *gidju*within crown Casuariiformes is supported by 13 unambiguous apomorphies distributed in several skeletal elements such as skull, sternum, synsacrum and femur	26	56	SN (26, 13, 10)

The stem-galloanserine *Asteriornis maastrichtensis* from the Latest Cretaceous Europe (Field et al., 2020) calibrated the root of our tree. As the skull and legs of *A*. *maastrichtensis* share exclusive derived characters with Galliformes and other Neognathae (Crane et al., 2024), it provides a minimum age for the split Neognathae and Palaeognathae split around 66 Ma. The soft upper bound was set at 86.5 Ma, which represents rich avian fossil deposits that completely lack neornithine representatives (Prum et al., 2015).

The remaining four fossil specimens calibrated nodes within Palaeognathae. *Palaeophasianus meleagroides* from Eocene North America (Wetmore, 1933) is among the oldest representative of Geranoididae, a family of long-legged birds sharing derived characters with the younger European Palaeotididae and the Asian Eogruidae and Ergilornithidae (Mayr, 2016b; 2019; 2022). As those three families are consistently placed on the ostrich stem, *Palaeophasianus meleagroides* provides a minimum age for the Palaeognathae root. The only known older stem palaeognath is *Diogenornis fragilis*, a specimen from Paleocene South America with uncertain affinities (Noriega et al., 2017; Mayr, 2022).

The second Palaeognathae internal calibration was provided by *Opisthodactylus horacioperezi* from Miocene South America (Agnolin and Chafrat, 2015). This oldest crown Rheidae genus shares with the extant *Pterocnemia* a derived hindlimb morphology (Agnolin and Chafrat, 2015; Noriega et al., 2017). This fossil prior was set between the two extant Rheidae genera *Rhea* and *Pterocnemia*.

The third internal palaeognath calibration was the fragmentary coracoid MACN-SC-3610 from Miocene South America representing the oldest unambiguous crown Tinamidae. Despite its fragmentary condition, cladistic analyses confidently place the specimen within Tinamidae as sister to the genus *Crypturellus*. This placement is supported by unique derived characters (Bertelli, 2017; Almeida et al., 2021). Other specimens found in the same age and locality have an unstable position (Bertelli et al., 2014). We used MACN-SC-3610 to calibrate the split between *Crypturellus* and its sister genus *Tinamus*.

Finally, the last internal palaeognath calibration was *Emuarius gidju* from Miocene Australia (Boles, 1992; 2001). Cladistic analyses using 25 ingroup taxa and 179 osteological characters firmly placed *E*. *gidju* within crown Casuariiformes supported by 13 unambiguous apomorphies distributed in several skeletal elements including the skull, sternum, synsacrum and femur (Worthy et al., 2014).

### 2.3 Divergence time estimates and fossil calibration strategies

The divergence times were estimated separately for the MTG, TKZ and PRM datasets using the PAML 4.10 package (Yang, 2007). The mean substitution rate estimated with BASEML was set as the Dirichlet-gamma prior. The absolute divergence times were estimated with the Bayesian program MCMCTree using a time unit of 1 million years and the uncorrelated relaxed clock under the GTR model with alpha = 0.5 and five Gamma categories. The parameters for birth, death, and sampling were 0.1. The σ^2^ prior was set to G (1, 10) for the MTG dataset, indicating moderate violation of the clock, whereas it was G (1, 0.1) for all the nuclear datasets indicating a higher violation of the clock as expected with thousands of independently evolving markers. Branch lengths and model parameters were estimated with the approximate likelihood method, designed to handle large phylogenomic data (Reis and Yang, 2011).

Substitution saturation in the MTG alignment was assessed using DAMBE7 (Xia, 2018). The proportion of invariant sites under a Poisson + invariant distribution model was estimated and added as a parameter of the substitution saturation test for all fully resolved sites (Xia et al., 2003; Xia and Lemey, 2009). Under default settings, saturation is likely if the observed index of substitution saturation (Iss) exceeds the critical threshold (Iss.c). For a symmetrical tree topology, such as the consensus phylogeny of Palaeognathae recovered from multiple genomic studies (see Introduction and Figure 1), the observed Iss was 0,4539, well below the Iss.c of 0,8155, suggesting minimal saturation of the MTG dataset. The observed Iss was significantly smaller even considering an for an extremely asymmetrical (and generally very unlikely) tree (Iss.c = 0.5709). To further validate this result, we plotted the uncorrected pairwise genetic distances (p-distances) against the model-corrected distances (JC69) for each mitochondrial partition using R (R Core Team, 2024) and the ape (Paradis and Schliep, 2019) and phangorn (Schliep, 2011) packages. The plots showed strong linearity with coefficients of determination (*R* ^2^) greater than 0.967 which, taken together with the substitution saturation test, provide robust evidence for minimal saturation in our MTG dataset.

The MTG topology was produced using a constrained backbone (order-level), and terminal relationships, branch lengths, partition scheme and substitution models were estimated with IQTREE 2.1.4 (Minh et al., 2020). The topology and the best partition scheme with ten partitions were used for molecular dating. The tree topologies for the nuclear datasets were constrained as well following the consensus backbone (Figure 1). The TKZ dataset was partitioned by molecular type (CNEE, C12 and UCE), and in addition each datatype was analyzed separately. Lastly, the PRM dataset was analyzed with 75 partitions as in the original study (Table 1).

The MCMC chain length, sampling frequency, and burn-in were optimized for each dataset to meet the statistical requirements of the data size. For the MTG dataset, the chain length was set to 20,000 iterations, sampling every 100, and a burn-in 10,000. For the TKZ dataset, the chain length was set to 100,000 iterations, sampling every 5,000, and a burn-in of 5,000,000. For the PRM dataset, the chain length was set to 20,000 generations, sampling every 100, and a burn-in 10,000. For the separate nuclear C12 we ran 50,000 iterations, sampling every 500, and set the burn-in to 10,000; for CNEE we ran 80,000 iterations, sampled every 2,000, and set the burn-in to 800,000 and for the UCE data we ran 50,000 iterations, sampled every 1,000, and burn-in 20,000. For the PRM dataset we ran 2,000 iterations, sampling every 500, and set the burn-in to 1,000. Two independent MCMC runs were performed for each dataset, with convergence verified in both. The ESS parameters were verified in Tracer 1.7.2 (Rambaut et al., 2018) and all values reached >200.

To assess whether age estimates of crown Palaeognathae are more influenced by internal fossil constraints or phylogenomic data composition, we developed two calibration strategies. The first included all the fossil priors that were available for all datasets (Table 2). All fossil calibrations were applied to all datasets, except for the prior for crown Rheidae in the PRM dataset as this node was represented by only a single species. The probability distribution for the fossil age uncertainty followed a skew-normal curve calculated with the SN package (Azzalini, 2023) for R (R Core Team, 2024). To accommodate the uncertainty of the Neornithes root age, the maximum age of all internal fossil priors was extended to 70 Ma. In the second strategy, all Palaeognathae fossils were removed, leaving only the *Asteriornis maastrichtensis* prior at the root (Table 3). All the time trees generated in this study, namely, MTG, TKZ, C12, CNEE, UCE, and the PRM with all fossil priors and root prior only are available as Supplementary Material S2. Furthermore, the differences in divergence time estimates and taxon sampling between the MTG and TKZ topologies are displayed in pdf format as Supplementary Material S3.

**TABLE 3:** Comparison of divergence times calculated with different calibration strategies for each dataset. Full Set strategy includes all fossils whereas Root Only excludes all ingroup (Palaeognathae) fossils. Specimen, age and phylogenetic placement of each prior as in Table 2. Time units are million years ago (Ma) and Confidence Interval is the 95% Highest Posterior Distribution (HPD) are rounded up to the next decimal place.

Dataset	Node	Calibration strategy			
		Full set		Root only	
		Mean	95% HPD	Mean	95% HPD
MTG	Palaeognathae	68	62–74	74	63–85
TKZ		62	56–68	80	71–86
PRM		62	56–67	42	35–49
MTG	Notopalaeognathae	63	57–69	68	57–79
TKZ		59	53–66	76	67–84
PRM		56	51–61	38	31–44
MTG	Non-Rheidae Notopalaeognathae	61	56–67	66	56–77
TKZ		56	48–63	72	62–81
PRM		55	50–60	37	31–43
MTG	Casuariiformes + Apterygiformes	56	48–63	58	43–72
TKZ		54	46–61	70	60–80
PRM		54	50–59	37	31–43
MTG	Casuariiformes	26	22–30	16	11–22
TKZ		33	24–43	30	11–52
PRM		13	11–15	6	4–7
MTG	Tinamiformes + Dinornithiformes	57	51–63	61	51–71
TKZ		47	38–56	60	48–71
PRM		–	–	–	–

Mean ages and confidence interval (95% HPD) are given in million years ago (Ma) for the major nodes in Palaeognathae and the neornithine root. The estimates were generated with a diverse array of phylogenomic data types (for details see Table 1).

## 3 Results

**FIGURE 2:**
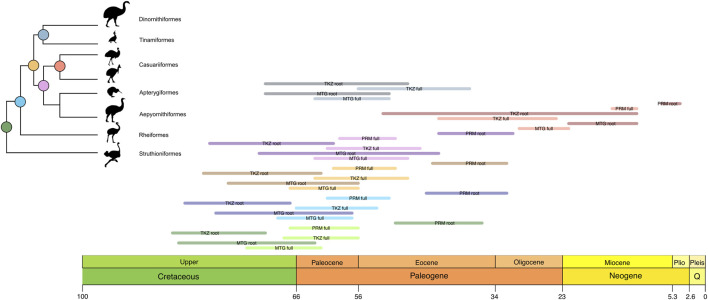
Differences in the confidence intervals of the ages estimated for each major Palaeognath clade and the Neornithine root using the full calibration set in this study and other major works (Table 4). The colors in the node circles match the colors on the horizontal bars, namely, red for Neornithes; green for Palaeognathae; blue for Notopalaeognathae; orange for non-rheid notopalaeognaths; pink for Casuariiformes + Apterygidae and Aepyornithidae (sampled only in the MTG dataset); coral for Casuariidae; and grey for Tinamiformes + Dinornithiformes. Each bar corresponds to one dataset or previous study.

**FIGURE 3:**
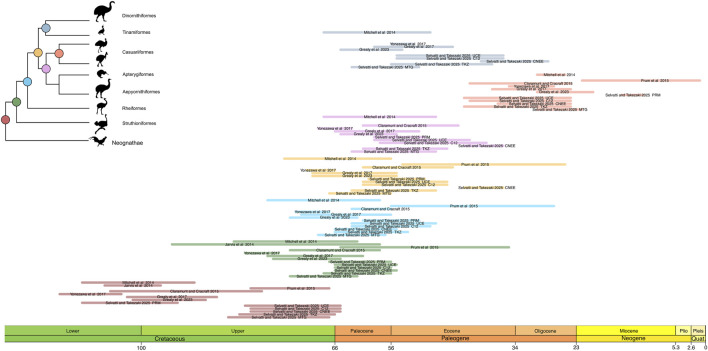
Results from varying the calibration schemes found in this study. The colors refer to the same nodes as in Figure 2. Light colors reflect the first calibration strategy (all fossils, as in Figure 2), whereas dark hues reflect the second calibration strategy (root only). Aepyornithidae was sampled only in the MTG dataset. The confidence intervals were extracted from Table 3.

We investigated the impact of internal calibration priors on divergence time estimates for the Palaeognathae radiation. Specifically, we assembled a diverse phylogenomic dataset to test whether the Eocene age (Prum et al., 2015) was caused by data type or a calibration strategy that lacked fossil priors outside Neognathae. Our resulting matrices (Table 1) comprised mitogenomes (MTG, 31 species, 14,307 bp, 10 partitions) and distinct nuclear genomic regions from different studies (TKZ, 14 species, 11,187,881 bp, 3 partitions; PRM, 9 species, 394,684 bp, 75 partitions). Each partition from the TKZ dataset was also analyzed separately, resulting in CNEE with 14 species, 4,504,498 bp, 1 partition; C12, 13 species, 4,797,876 bp, 1 partition; UCE, 14 species, 1,885,507 bp, 1 partition. Our posterior probability estimates with MCMCTree indicated that the different cellular compartments and genomic regions sampled in our datasets have markedly different substitution rates. Specifically, the mean substitution rate averaged for the ten partitions of MTG was the highest (*µ* = 0.01107/million years [Myr]), followed by the rate averaged for the 75 partitions of PRM (*µ* = 0.00091/Myr) and finally for the rate averaged for the three partitions of TKZ (*µ* = 0.00053/million years). The divergence time estimates for each dataset using our full calibration set are provided in Table 4, which also summarizes estimates from major previous studies focusing on either Palaeognathae or, more broadly, Neornithes. The confidence intervals from Tables 3 and 4 were used to generate Figures 2, 3, respectively.

**TABLE 4:** Mean ages and confidence interval (95% HPD) of the nodes estimated in this study and in other recent phylogenomic studies.

Node	Study																									
	Selvatti and Takezaki 2025 (MTG)		Selvatti and Takezaki 2025 (TKZ)		Selvatti and Takezaki 2025 (CNEE)		Selvatti and Takezaki 2025 (C12)		Selvatti and Takezaki 2025 (UCE)		Selvatti and Takezaki 2025 (PRM)		Grealyet al. (2023)		Grealyet al. (2017)		Yonezawaet al. (2017)		Claramunt and Cracraft (2015)		Prumet al. (2015)		Jarviset al. (2014)		Mitchellet al. (2014)	
	Mean	95% HPD	Mean	95% HPD	Mean	95% HPD	Mean	95% HPD	Mean	95% HPD	Mean	95% HPD	Mean	95% HPD	Mean	95% HPD	Mean	95% HPD	Mean	95% HPD	Mean	95% HPD	Mean	95% HPD	Mean	95% HPD
Neornithes	76	67–85	74	66–83	72	65–81	72	65–81	72	65–82	102	94–111	94	89–102	95	87–103	110	104–115	91	79–106	72	67–81	101	97–107	101	91–111
Crown Palaeognathae	68	62–74	62	56–68	61	55–66	61	56–66	60	55–66	62	56–67	70	77–65	69	61–78	79	76–82	65	58–74	50	35–65	83	58–95	72	62–84
Notopalaeognathae (MRCA of Rheiformes, Tinamiformes, Casuariiformes and Apterygiformes)	63	57–69	59	53–66	61	55–66	56	49–63	55	48–63	56	51–61	66	74–62	64	56–72	70	67–73	57	56–63	43	27–56	–	–	68	58–78
MRCA of Tinamiformes, Casuariiformes and Apterygiformes	61	56–67	56	48–63	39	36–43	53	46–61	54	46–61	55	50–60	62	70–55	62	55–70	69	66–71	55	45–61	41	25–54	–	–	65	56–75
MRCA of Casuariiformes and Apterygiformes	56	48–63	54	46–61	39	36–43	49	39–58	51	42–59	54	50–59	60	55–65	58	51–66	66	63–69	54	44–61	–	–	–	–	58	48–68
MRCA of Casuariiformes	26	22–30	33	24–43	30	24–37	32	24–42	32	24–43	13	11–15	27	20–30	31	24–38	31	27–35	28	24–42	9	1–22	–	–	26	25–30
MRCA of Tinamiformes and Dinornithiformes	57	51–63	47	38–56	34	28–40	45	36–55	45	36–55	–	–	59	65–54	51	45–59	53	52–56	–	–	–	–	–	–	58	49–68

Mean ages and confidence interval (95% HPD) of the nodes estimated in this study and in other recent phylogenomic studies.

The divergence times estimated separately for each partition of the TKZ dataset remained very similar to the estimates for the concatenated matrix, indicating the consistency of our results (Figures 2, 3; Table 1). However, the separate analyses of the CNEE dataset produced short internodes in the palaeognath backbone, leading to extremely similar age estimates for two nodes. The first was between Struthioniformes and the remaining palaeognaths [Notopalaeognathae], and the second for the non-Rheiformes Notopalaeognathae [Tinamiformes, Apterygiformes and Casuariiformes] (Figure 2). The CNEE estimates were also in conflict with the other datasets within palaeognathae. For example, the estimates for the non-Rheiformes Notopalaeognathae, the split between Apterygiformes and Casuariiformes and between Dinornithiformes and Tinamiformes were younger (39–30 Ma) compared with the estimates from the remaining datasets (61–45 Ma), with little to no overlap between the confidence intervals (Figure 2; Table 4). The single exception was the emu-cassowary node (Casuariiformes), which estimates with the CNEE dataset remained similar with greatly overlapping confidence intervals with the other datasets (Figure 2; Table 4).

The phylogenomic datasets were used to estimate the divergence times with five fossil priors (one outgroup and four ingroup; Table 2) under two distinct calibration strategies. The first included all fossil priors, while the second used only the root fossil prior, testing the effect of excluding ingroup fossil calibrations across different data types. The results are summarized in Figure 3 and Table 3 and detailed as follows. In the first calibration strategy, the mean divergence time estimates remained consistent, with less than 10 Myr difference across datasets, though estimates for MTG tend to be slightly larger than those for nuclear TKZ and PRM and the difference between estimates for Casuariiformes were larger (Figure 3; Table 3, light colors). The oldest mean estimates for the MRCA of crown Palaeognathae was obtained by the MTG dataset at 68.1 Ma (95% Highest Posterior Distribution [HPD] = 62–74.2 Ma), followed by the very similar ages of the TKZ at 62.4 (56.4–68.3 Ma) and PRM at 62.4 (56.7–67.2 Ma) datasets. Furthermore, the confidence intervals overlapped greatly with an average variation of 12 Myr between minimum and maximum for each estimate. In the second calibration strategy, however, the results differed markedly (Figure 3; Table 3, dark colors). Mean ages were older in the TKZ (12 Myr) and MTG (6 Myr) datasets compared to the first strategy. In contrast, the PRM dataset yielded a much younger mean age (−20 Myr), strikingly deviating from the estimates of the first strategy. Furthermore, the mean ages varied extensively among datasets. The oldest mean age was produced by the TKZ dataset (80 Ma), followed by the MTG (74 Ma) and PRM datasets (42 Ma). In the second calibration strategy the confidence intervals were large and varied considerably across datasets, averaging 18 Myr and with notable gaps between estimates denoting conflicting results (Figure 3). For example, although the intervals between the MTG and TKZ estimates showed substantial overlap indicating a higher degree of agreement, they did not overlap with the confidence interval of the significantly younger PRM estimate (Figure 3).

The divergence times of the internal palaeognath clades remained consistent across different datasets in our results, with the already mentioned younger estimates within Notopalaeognathae in the CNEE dataset alone and in the PRM dataset (Figure 2; Table 4). The mean age of the MRCA of the Notopalaeognathae was dated between 56 and 63 Ma, and the clade that contains the non-Rheidae Notopalaeognathae was dated around 55–61 Ma (39 Ma in CNEE). The estimates for the MRCA of Casuariiformes and Apterygiformes (54–56 Ma; 39 Ma in CNEE) were similar to the estimates for the Tinamiformes and Dinortnithiformes MRCA (47–57 Ma; 34 Ma in CNEE). However, the estimates using the PRM dataset were consistently younger for the Casuariiformes. Although this node was dated between 26 and 33 Ma with the MTG, TKZ, C12, CNEE, and UCE datasets, the estimate with the PRM dataset was 13 Ma, which is similar to the age estimated in the original study (9 Ma; Prum et al., 2015). However, in that study the confidence interval was substantial (1–22 Ma), whereas it was significantly smaller in our results (11–15 Ma; Figure 2). In the second calibration strategy, the results showed significant disparities, especially for the deep nodes in the nuclear dataset. Under this strategy, for instance, the estimates from TKZ produced virtually no overlap with the confidence intervals of the same dataset when all calibration were included (Figure 3; Table 3). That same disparity between the calibration strategies were present but less pronounced in the MTG estimates. Nevertheless, regardless of calibration strategy, the confidence intervals of estimates with TKG and MTG overlap consistently. However, a remarkable trend was observed regarding the PRM dataset. In the second calibration strategy, the estimates for all the nodes except Casuariiformes produced older age estimates in the MTG, TKZ, C12, CNEE, and UCE dataset, however, the PRM dataset always resulted in younger estimates compared with the first strategy (Figure 3; Table 3).

## 4 Discussion

Palaeognaths are one of the most peculiar avian lineages, with an enduring enigmatic evolutionary history and biogeography (Mayr, 2011; Widrig and Field, 2022). Recently, thorough scrutiny of phylogenomic data advanced our knowledge on the branching order in palaeognaths, reducing some of the conflicts regarding tree topology (Yonezawa et al., 2017; Takezaki, 2023). Nevertheless, crown Palaeognathae age estimates have varied significantly over the last decade (Figure 1; Table 1), from the Middle Cretaceous (97 Ma; Haddrath and Baker, 2012) to the Eocene (51 Ma; Prum et al., 2015). As more loci, lineages and fossils were analyzed, most phylogenomic data converged on a crown Palaeognathae age around the K–Pg boundary (66 Ma), but no Eocene age was recovered a second time by independent data. That disparity might result from different genomic sampling and fossil calibration strategies, including number, position, and age prior probability, which hinder direct comparison among those studies. Consequently, pinpointing the cause for such conflict remained inconclusive, leaving the age of the crown group Palaeognathae one of the most elusive aspects of the Palaeognathae complex evolution.

We analyzed a large diversity of phylogenomic data, including mitogenomes and nuclear coding and non-coding loci, and the original data that recovered the Eocene age for crown palaeognaths. Importantly, the taxon sampling varied considerably between datasets, with the MTG scoring the highest number of tips (31 species), and represents the largest mitogenomic dataset to date for which divergence times were estimated. This dataset nearly doubles the sample size of Grealy et al. (2017) and exceeds that of Grealy et al. (2023) by eight species. Divergence times were also estimated for nuclear genomic data from 14 species, thoroughly curated to minimize saturation (Takezaki, 2023). Thus, our sampling encompassed taxonomic diversity and millions of base pairs across distinct genomic regions and cellular compartments. In addition to controlling for taxonomic and genetic diversity, we tested whether conflicts in the Palaeognathae root age might result from incomplete placement of fossil calibrations, particularly within the ingroup. We applied two calibration strategies, the first using all available fossils (Table 2) and the second excluding ingroup calibrations.

Our results show remarkable consistency across different datasets despite varying taxon sampling and molecular data type, especially for the root age of crown Palaeognathae, which was the focus of this study. The estimates of most datasets also remained consistent for the estimates for the internal nodes except for the CNEE data analyzed separately. The estimates from this dataset produced very short internal branches in the Palaeognathae backbone, and the estimates for the non-Rheidae Notopalaeognathae nodes were significantly younger than our estimates and compared to other studies (Table 4). Those results might be explained by the notably slow evolution of CNEEs, which is below the neutral rate (Edwards et al., 2017). Previous research shows that even after removing loci with evidence for phylogenetic noise (e.g., recombination and long branches), the CNEE tree produced very short internodes in the palaeognath stem (Takezaki, 2023), which coincide with the collapsed branches in our CNEE time tree. These results suggest that this particular CNEE dataset is challenging to estimate the details of the divergence times for nodes between the root and family-level Palaeognathae clades. In contrast, however, the estimates for the Casuariiformes node remained consistent across datasets including the CNEE except for the PRM which produced a much younger estimate (Table 4). This discrepancy of the PRM may be due to the differences in taxon sampling or that it consists mostly of coding sequence including third codon positions. Interestingly, this node received an explicit fossil-based calibration, which might consist of an exceptionally informative prior for this particular node. Future studies focusing on the CNEE power to estimate the divergence times with confidence for Palaeognathae and other avian lienages will be of great interest to clarify our findings.

Our main result is that divergence time estimates in Palaeognathae are profoundly influenced by calibration strategy (Tables 1 and 3). Empirical studies have demonstrated that genomic regions evolve at different rates. For example, genes that protein coding, sex-related, or mediate defense response have very high substitution rates (Moutinho et al., 2020). Thus, as different genomic regions and cellular compartments evolve at distinct rates (Mendes and Hahn, 2016), we expected that the observed conflicting estimates were caused by studies sampling different genomic regions. Due to the heterogeneous nature of the sampled genomic regions and their differing evolutionary patterns, we anticipated variation in substitution rates, which was confirmed by our findings (see Results). However, despite substitution rate heterogeneity, all phylogenomic datasets converged to similar ages and confidence intervals when the full set of fossil priors were used (Table 2). In contrast, when no ingroup calibration was used, time estimates varied immensely, often with irreconcilable confidence intervals (Figure 3; Table 3). This is somewhat surprising as empirical and simulation studies showing that calibrations set near or at the root yield precise and accurate divergence time estimates (Duchêne et al., 2014; Mello and Schrago, 2014).

Our results indicate that root calibration alone may be insufficient for groups with complex evolutionary history such as Palaeognathae. Empirical data and simulations *in silico* show that multiple ingroup calibrations help reduce disparities caused by differences in genetic distance between nodes with and without calibrations (Rutschmann et al., 2007; Marshall, 2008; Reis et al., 2018). Our results showed that conflicting ages and confidence intervals emerged from the same dataset with different calibration strategies, while adding more internal calibrations improved the precision of the estimates. Furthermore, previous studies have shown that multiple calibrations improve age estimation accuracy (Linder et al., 2005; Villaverde et al., 2021), a trend we also observed, where the use of multiple fossils resulted in more consistent divergence time estimates.

Our findings further reinforce prior evidence that the use of a single fossil calibration at the root of a tree is insufficient for reliable divergence time estimation within the ingroup, in cases when a long, uncalibrated stem branch separates the root from the first ingroup split. In the case of palaeognaths, the deep divergence between Neornithes and the crown palaeognath ancestor spans tens of millions of years, making it difficult for a relaxed clock model to accommodate rate heterogeneity across such a wide temporal range. In this context, a single calibration might fail to adequately constrain the rate estimates within the ingroup, leading to inflated or biased node age estimates. In a broad analysis of Bovidae, multiple fossil calibrations distributed across the tree consistently improve temporal accuracy and reduce uncertainty (Bibi, 2013). Furthermore, recent simulation-based assessments demonstrate that when evolutionary rates vary substantially across lineages, commonly used clock models including uncorrelated and autocorrelated relaxed clocks may perform poorly, particularly in the absence of internal calibrations (Hagemann et al., 2023). Although local clock models may be better suited to handle such heterogeneity, they are not implemented in MCMCTree and remain difficult to apply to genomic-scale datasets due to model complexity and computational limitations. Nonetheless, our results highlight the practical importance of using multiple, well-placed fossil calibrations to overcome limitations associated with model rigidity and clock non-uniformity.

Previous studies that used multiple calibration priors overlap partially with our set of fossils. Specimens of the genus *Emuarius* and the specimen *Opisthodactylus horacioperezi* have been used as minimum dates for the Casuariiformes and Rheidae clades, respectively (Table 1). As those fossils have clear apomorphies shared with their respective clades and their phylogenetic position have been corroborated by formal cladistic analyses, they indeed satisfy the recommended best practices for molecular dating prior design based fossil specimens (Parham et al., 2012). However, as the following three fossil specimens satisfy only some of the best practices, they were not to used in the present study. The Brazilian fossil *Diogenornis fragilis* was originally assigned to a rheiform assembly from the Miocene (Alvarenga, 1983; Mayr, 2016a). However, those similarities might reflect plesiomorphies and its phylogenetic position remains poorly resolved, and *Diogenornis* is considered a stem rheiform at best (Mayr, 2016a; Widrig and Field, 2022). Because stem fossils share only some characteristics with the extant (crown) clade, they provide little information for the split that originated the crown clade and thus should be avoided as calibration priors. This justification is extended to the kiwi-like fossil *Proapteryx* (Worthy et al., 2013). As it shares only some of the derived characters that define the extant Apterygiformes, it is considered part of the kiwi stem Widrig and Field, 2022) and thus unsuitable to calibrate the crown Apterygiformes. Finally, the remaining differences in our prior choice regards the Tinamiformes fossil record. In the context of molecular dating, fossil ages provide a minimum date for the clade which it belongs with confidence (Ho and Phillips, 2009). Therefore, priority should be given for the specimens with the oldest geological age and with unambiguous phylogenetic placement (Ho and Phillips, 2009; Parham et al., 2012). Although the specimen MLP 87-XI-20-3 from the La Pampa Province (Argentina) is undoubtedly part of the Tinamiformes, it does not represent the oldest record in that clade (Bertelli et al., 2014). That status belongs to the MACN-SC-1399 and AMNH FAM 9151 specimens from the early Miocene Argentinian Formations of Pinturas and Santa Cruz, respectively. Nevertheless, those fragmentary specimens have a highly unstable position in cladistic studies (Bertelli, 2017; Almeida et al., 2021). The specimen MACN-SC-3610 is from the same age and locality of the specimen MACN-SC-1399, but with much higher confidence in its phylogenetic placement (Bertelli et al., 2014), thus justifying our choice as our fossil prior within Tinamiformes.

Estimating the divergence time of the crown Palaeognathae ancestor with both consistency and precision is crucial for resolving conflicting estimates and ensuring that hypotheses for early Palaeognathae evolution are developed reliably. Three competing diversification hypotheses for the group are currently supported. The first assumes that the crown Palaeognathae originated as an ancient lineage that existed between 83 and 72 Ma in the Upper Cretaceous (Haddrath and Baker, 2012; Jarvis et al., 2014; Yonezawa et al., 2017). Those estimates predate all known definitive neornithine fossils (Mayr, 2016a), and that ancestral stock with several large-bodied descendants would have left no trace in the fossil record, which is rich for many other non-noernithine avian clades (Mayr, 2016a). In the studies that support the first hypothesis, the antiquity of the palaeognath root also reverberates in the internal nodes (Table 4), implying a burst of palaeognathae diversification prior to the K–Pg. In this context, the Upper Cretaceous hypothesis favors the classic view in which cladogenesis within palaeognathae is linked with the fragmentation history of Gondwana, where flightlessness evolved once in the ratite (Palaeognathae except Tinamiformes) ancestor before the fragmentation of the supercontinent, and modern ratite lineages are the product of the subsequent continental breakup (Cracraft, 1973). At that time, the fragments of the supercontinent remained in close proximity (McLoughlin, 2001; Chatterjee et al., 2012; Ezcurra and Agnolin, 2012), potentially facilitating terrestrial biotic interchanges that would have included flightless ratites. However, this classic hypothesis also hinges on ratite monophyly, which is rejected by molecular and embriological data (Widrig and Field, 2022). Furthermore, our results suggest that this hypothesis may be influenced by limitations in the number and position of the fossil calibration priors. For instance, in our study, all ages that are older than our oldest estimate (74 Ma maximum 95% HPD of the MTG dataset) used one or zero fossil priors for the ingroup palaeognath clade (Tables 1 and 4).

In the second hypothesis, the crown group palaeognaths would be considerably younger and long after Gondwana break up (Mitchell et al., 2014; Claramunt and Cracraft, 2015; Grealy et al., 2017; 2023; Stiller et al., 2024; Wu et al., 2024). In this scenario, the crown Palaeognathae ancestor split into the Struthioniformes and Notopalaeognathae around the K–Pg boundary. After the divergence of Struthioniformes ancestor from the Palaeognathae root, the splits that originated the Notopalaeognathae orders occurred within the Paleocene and the Early Eocene. During that time, many continents in the Southern Hemisphere were still partially connected, such as Australia, Antarctica, South America and Zealandia, allowing the ancestral stocks of the clades that are endemic to those specific landmasses to disperse and achieve their present-day distributions (Claramunt and Cracraft, 2015; Yonezawa et al., 2017; Takezaki, 2023).

The third hypothesis suggests an Eocene age for crown Palaeognathae (Prum et al., 2015). In that timeframe, the extant clades would have split near the transition to the Oligocene or even later (Tables 3 and 4), a period by which the Southern landmasses were already far apart. The Eocene age hypothesis demands extremely long-distance dispersals by volant ancestors within each palaeognath lineage, which is currently unsupported by the fossil record and the biology of the extant species. For instance, although tinamous are volant, they are essentially ground-dwelling birds that only fly when needed, and their flight is a short-distance burst unlikely to sustain overwater dispersal (Widrig and Field, 2022). The only candidate palaeognaths with potent flight capabilities are the Lithornithidae, a Paleocene group with some derived traits shared with extant palaeognaths (Widrig and Field, 2022). In the Eocene age hypothesis, all Paleocene Lithornithidae clearly fall outside the crown Palaeognathae, thus ruling out the possibility of placing certain specimens within internal branches such as Tinamidae, as suggested by some morphological characters and character weighting (Worthy et al., 2017).

Our results support the second hypothesis and consistently reject the other two (Figures 2, 3; Tables 3 and 4). We provide empirical evidence that robust divergence time estimates for Palaeognathae are achieved by combining broad taxonomic and genomic diversity with multiple fossil calibrations, including both the root and ingroup. This strategy yielded consistent and precise results, which improved reliability across different data types and calibration strategies. Furthermore, the variation present in the different molecular data types reflected a broad range of evolutionary rates (see Results). The PRM dataset consists mostly of (80%) coding regions (Reddy et al., 2017), which have shown in previous studies to be notoriously challenging to extract phylogenetic signal, producing unstable, weakly supported and incongruent results (Chen et al., 2017; Reddy et al., 2017). Therefore, although the PRM dataset provides a comprehensive sample of neornithine clades, our findings suggest that the genomic regions sampled might not offer sufficient or reliable signal for estimating the split times in Palaeognathae without calibration priors on the internal nodes. Even with our full calibration set, while the PRM dataset estimates aligned more closely with those of other datasets, younger ages continued to be observed for shallow nodes like Casuariiformes (Figure 2). Specifically, while other datasets estimated the age of Casuariiformes to be in the Oligocene (22–43 Ma), the PRM-based estimates remained within the Miocene (15–11 Ma), albeit with a narrower confidence interval compared to the results of the calibration strategy used in the original study (Figure 2; Table 4). As the same fossil calibration strategy was applied to different datasets converged on consistent estimates, we conclude that the age of crown Palaeognathae is likely more influenced by the number and position of fossil constraints than by molecular data type, or a combination of both. Previous studies show that variations in age estimates in other clades can significantly influence biogeographic and ancestral trait interpretations in major diversification events in passerines (Selvatti et al., 2015) and in turtles (Selvatti et al., 2023). Our results also align with recent genome-wide time estimates for avian evolution and neornithine diversification (Stiller et al., 2024), attesting the robustness of our time trees.

Our results indicate that molecular data type alone cannot explain the reported Eocene age for crown palaeognaths (Prum et al., 2015). Our re-analyses with the same dataset using the original fossil calibration strategy (without ingroup fossil calibrations) produced identical results. This contrasts sharply with estimates from the other datasets, which were older in the absence of ingroup fossil constraints. However, when multiple internal fossil calibrations were applied to the original PRM dataset, the results closely aligned with those from the other genomic datasets sampled here and in previous studies (Table 1). The study by Prum et al. (2015) is a landmark in avian phylogenomics, being the first to include a broad taxon sampling, more than three times that of the pioneering work by Jarvis et al. (2014). However, the exclusion of fossil priors for Neognathae nodes may have influenced the divergence time estimates for the Palaeognathae root. Consequently, we propose that the Eocene age for Palaeognathae could reflect the combined effect of the absence of fossil calibrations at and near the neornithine root, as well as within the Palaeognath ingroup.

Our time-calibrated phylogeny allows for the possibility that some of the oldest Palaeognathae fossils may indeed belong within the crown Palaeognathae clade, whereas this possibility is excluded under the Eocene divergence hypothesis. For instance, in the K–Pg age hypothesis supported here, at least some Lithornithidae might be assigned to internal branches, providing explicit support even for hypotheses based on long-distance dispersals. A palaeognath radiation around the K–Pg requires at least some degree of overseas dispersals, thus compatible with a volant ancestor that might have included the Lithornithidae. The same principle applies to another key Paleocene fossil, *D. fragilis*. This ancient, flightless South American bird is one of the oldest named Palaeognathae (Widrig and Field, 2022). Despite ongoing debate regarding its affinity with either Casuariiformes or Rheiformes, assuming an Eocene origin for crown Palaeognathae precludes *Diogenornis* from being considered part of the crown group, necessitating its placement as a Palaeognath stem—an assignment for which there is no morphological support. In contrast, if the crown Palaeognathae originated around the K–Pg boundary, as suggested here, the affinities of *Diogenornis* clearly align with the crown Palaeognathae, thereby reducing the uncertainty surrounding its phylogenetic placement.

Although our results emphasize the critical role of internal fossil constraints in stabilizing divergence time estimates, we acknowledge that deep branches, such as the root of Palaeognathae and Neornithes, may remain sensitive to unmodeled rate variation or substitutional saturation. While saturation tests and data filtering indicated no saturation, and reanalysis of the Prum et al. (2015) dataset showed that replacing a broad neognath outgroup with *Gallus* alone had no effect on age estimates within Palaeognathae, we acknowledge that outgroup configuration could still influence branch length modeling under different data and age constraints. Future work should systematically assess the impact of alternative or expanded outgroup sampling on rate estimation and divergence dating while still verifying the effects of ingroup fossil constraints, particularly in datasets spanning deep evolutionary timescales.

## 5 Conclusion

Time is a fundamental aspect of evolution, as diversification hypotheses depend on knowing when lineages originated within specific geological contexts. In summary, our results demonstrate that multiple internal fossil constraints have a greater impact on crown Palaeognathae age estimates than different phylogenomic data types. The age of the crown Palaeognathae was consistently estimated to fall between the Latest Cretaceous and Earliest Paleogene (68–62 Ma), supporting the hypothesis that the group originated around the K–Pg boundary rather than in the Eocene. This reduces uncertainties surrounding the origin of the extant lineages and aligns with the phylogenetic placement of Paleogene fossils, such as *Diogenornis* (which was not included in our calibration), within the crown Palaeognathae. Our time tree also provides a clearer understanding of the relationships between crown Palaeognaths and the extinct Lithornithidae. Although some Lithornithidae species have been considered stem members of Palaeognathae (Mayr, 2022; Widrig and Field, 2022), their phylogenetic placement depends on the estimated age of the crown group. Assuming an Eocene origin for the crown Palaeognathae (Prum et al., 2015), these fossils would be placed outside the crown. If the crown Palaeognathae split is dated to the K–Pg boundary, some of these fossils could be positioned more internally within the Palaeognathae tree as stem members of major clades (Widrig and Field, 2022), shedding light on the intricate evolutionary history of this remarkable avian lineage, and highlighting the profound role of geological time in shaping biodiversity.

## Data Availability

Alignments generated in this study (MTG and TKZ) are available as Supplementary Material S1 deposited in Figshare (https://figshare.com/projects/Data_from_Internal_fossil_constraints_have_more_effect_on_the_age_estimates_of_crown_Palaeognathae_than_different_phylogenomic_data_type_/245348). The time trees (MTG, TKZ, C12, CNEE, UCE, and PRM with all fossil priors and root prior only) are available (newick format) as Supplementary Material S2. The differences in divergence time estimates and taxon sampling between the MTG and TKZ topologies are provided in pdf format in Supplementary Material S3. The original nuclear datasets analyzed in this study from Takezaki (2023) can be found at https://datadryad.org/stash/dataset/doi:10.5061/dryad.1jwstqjzs, and the original Prum et al. (2015) can be found at https://zenodo.org/records/28343.

## Glossary

Bayesian Relaxed Clock: A molecular dating method that estimates divergence times between lineages by allowing evolutionary rates to vary across branches and incorporating uncertainty in both rates and fossil calibrations.
Calibration Prior: A constraint based on (preferably) fossil or geological data used to convert the genetic differences into absolute (real) divergence ages.
Crown Group: The most recent common ancestor of all living members of a group and all its descendants.
Divergence Time: The point in evolutionary history when two species or lineages split from a common ancestor.
Fossil Constraint: A fossil used to inform the minimum (and sometimes maximum) age of a node in a phylogenetic tree, often with a probabilistic prior distribution.
Ingroup/Outgroup: In phylogenetics, the ingroup is the primary set of species under study, while the outgroup is a related lineage used for rooting the tree.
Mitogenome: The complete mitochondrial genome of an organism, often used in phylogenetic analyses for its maternal inheritance and relatively rapid evolution.
Molecular Clock: The classic 1965 hypothesis that predicted that genetic mutations accumulate at a constant rate, allowing estimates of divergence times based on genetic distance. The idea that substitution rates vary within and among genes, chromosomes, genetic compartments, and clades fostered the development of methods that take such variation and uncertainty into account.
Neornithes: The clade comprising all modern birds, including both Palaeognathae and Neognathae.
Node (in a phylogenetic tree): A point on a tree that represents a divergence event where one ancestral lineage splits into two or more descendants.
Notopalaeognathae: A clade within Palaeognathae that includes all members except the ostrich (Struthioniformes), thus comprising Rheiformes, Tinamiformes, Casuariiformes, Apterygiformes, and extinct relatives.
Partition Scheme: A strategy where sequence alignments containing different data subsets (e.g., gene regions or codon positions) are grouped based on common patterns such as base composition and rate heterogeneity. The best evolutionary model is then selected for each partition and proceed to phylogenetic tree inference.
Phylogenomics: The use of genome-scale data to reconstruct evolutionary relationships and histories.
Posterior Probability: A statistical measure in Bayesian analysis indicating the likelihood of a hypothesis (e.g., a divergence time) given the data and model.
Sequence Alignment: The process of arranging DNA, RNA, or protein sequences to identify regions of similarity that may indicate functional, structural, or evolutionary relationships.
Stratigraphy: The study of rock layers (strata) and their use in dating fossils and reconstructing geological history.
Substitution Rate: The speed at which genetic mutations become fixed in a lineage over time; a key parameter in tree reconstruction and molecular dating.
