# Supplementary figures and images for: Internal fossil constraints have more effect on the age estimates of crown Palaeognathae than different phylogenomic data type
Source: Front Bioinform. 2025 Aug 7;5:1563786. doi: 10.3389/fbinf.2025.1563786 (PMC12368558; doi:10.3389/fbinf.2025.1563786)

# MTG (mitogenomic concatenated dataset)

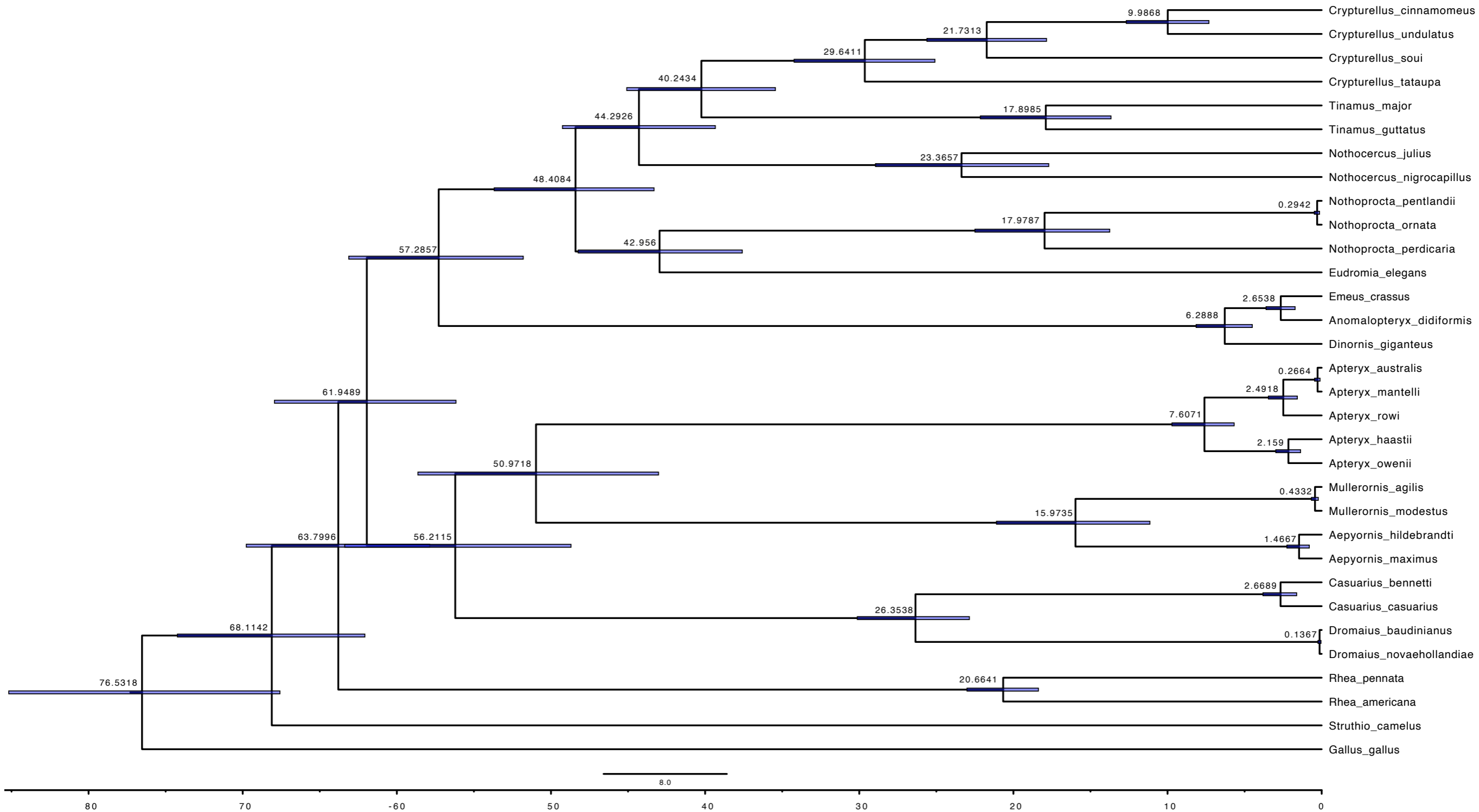

# TKZ (nuclear concatenated dataset)

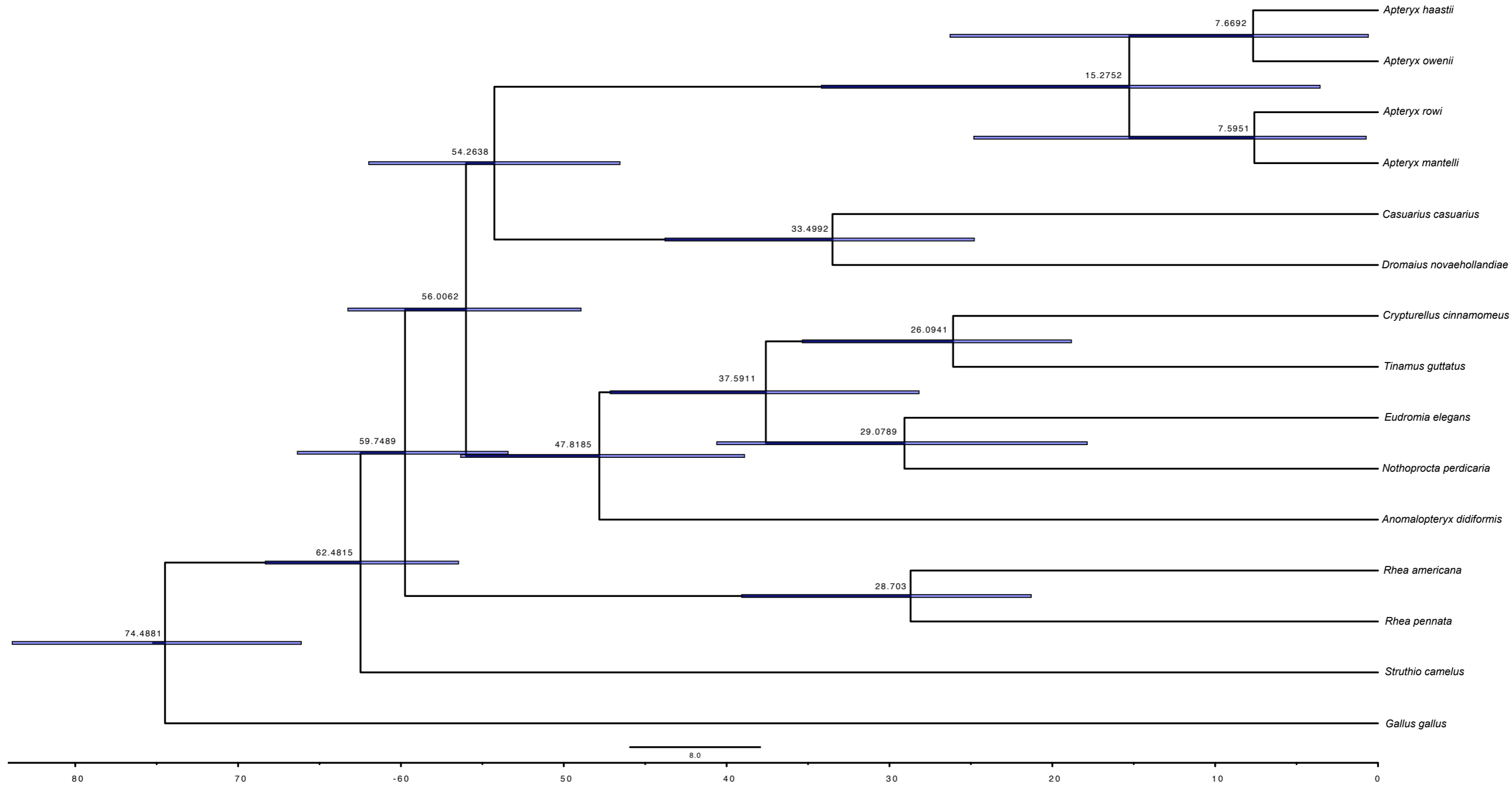

Supplement: Supplementary file 1 [file Presentation1.zip › SM S3 pdf trees.pdf]
